# Hospital Meetings, &c.

**Published:** 1899-05-06

**Authors:** 


### HOSPITAL MEETINGS, &C.

### ROYAL HOSPITAL FOR INCURABLES.

In the absence of the Duke of Cambridge, Mr. H. J. Allcroft, the treasurer of the institution, took the chair at the annual dinner of the Royal Hospital for Incurables, held in the Whitehall Rooms of the Hotel Metropole on Tuesday, 25th ult. In proposing the toast of the institution, the Chairman recalled the fact that this was the second occasion upon which he had filled that post; and also that the only other instance of the chair at one of their dinners being taken twice by the same individual was that of his own father, who had done so in 1872 and in 1880. Looking back over the history of the charity, he said that at their first election in 1854 there were only six patients; to-day they had 220 inmates of the hospital, and no less than 630 pensioners scattered all over the country. So flourishing was the state of their finances that at the election in May next they would be able to take in thirty-five—five more than in any other year except that of the Jubilee, when they elected fifty as a special recognition of the date. As an illustration of the magnitude to which they had grown he might put it that supposing a millionaire were to offer to give them sufficient to carry on their work on its present lines in perpetuity, the sum required to be invested in Consols would be nearly a million and a half sterling. They were, he was glad to say, passing through a very favourable year, but this was largely due to the number of legacies that had fallen in. Five years ago they had been obliged to borrow £5,000, but this had all been repaid, and they were free from debt. That their work could not be carried out without very considerable expense of a peculiar kind was shown by one item in that year's accounts. The cost of repairs alone to the wheeled chairs they provided for those patients unable to walk amounted to over £70. Their seaside house was another expensive item, but it was immensely appreciated. One of their best features, he thought, was that they were truly and thoroughly unsectarian. When candidates came before them they had necessarily to go through a most careful and detailed questioning to make sure that the case was a genuine one, but the one question never asked was: What is your religion? He maintained that there was no more deserving object of charity than the Royal Hospital for Incurables. During the course of the entertainment which followed the dinner, the secretary read the list of subscriptions and donations, amounting together to 1,500 guineas.

### EVELINA HOSPITAL FOR SICK CHILDREN.

The Hon. Conrad Dillon, chairman of the Committee of Management, presided at the twenty-ninth annual meeting of the Evelina Hospital for Sick Children on Wednesday, April 26th. In moving the adoption of the report, he expressed the deep sorrow of the committee at the sudden death of the founder and president of the hospital, Baron Ferdinand Rothschild, on December 17th last. Only a few days before his death he visited and inspected the hospital, entering with even more than his usual spirit into the various details of its management. From the date of the foundation of the hospital in 1869 he had always taken the closest interest in its welfare, and was kept constantly informed of all that took place within its walls, but at the same time he was always most anxious that it should secure the full attention of the governing body and not rely solely on himself. In view of the need for increased accommodation he lately purchased a plot of land adjoining the hospital, and placed £10,000 at the disposal of the committee for improving and extending the building. His bequest of £100,000 endows the hospital to the extent of his former annual subscription, but aside from the financial question the committee felt they had lost a helper and friend on whose sympathy and advice they could always rely. Miss Alice de Rothschild had very kindly paid the subscription which would have fallen due in January as a tribute to her brother's memory. The ordinary income for the year amounted to £4,674, while £2,175 was received from legacies. The ordinary expenditure was £5,490, extraordinary expenditure (including £1,000 invested and £500 transferred to deposit account) amounted to £1,961. During the year 985 children were treated in the hospital; 109 were sent to convalescent homes at the seaside and in the country. The out-patients numbered 7,633, and the number of attendances was just under 40,000. The report and accounts were adopted without discussion.

### LONDON LOCK HOSPITAL.

The 152nd annual meeting of the London Lock Hospital and Rescue Home was held on Thursday, April 27th, Mr. E. Parker Young presiding. The report presented showed receipts of £8,269, and expenditure £8,225, the outstanding debt of £1,258 being thus reduced by £43; £1,000 was also received through the Bishop of London from the Marriott Fund, and invested in accordance with the terms of the gift. During the year £973 was spent in repairs. The Chairman, in moving the adoption of the report, said they were making an appeal for £2,500 in order to put the drainage of the Harrow Road Hospital in a proper state. The Sub-Committee of the Prince of Wales's Fund had recommended that the sum of £500 be granted on condition that this work was at once taken in hand, and about £300 had been received in addition. The idea of holding a dinner this year had been abandoned. The report and accounts were adopted without dissent, and votes of thanks to Lord Kinnaird, the president, and to the medical staff concluded the proceedings.

